# Real-world precision medicine data in metastatic prostate cancer — a retrospective cohort study

**DOI:** 10.3389/fonc.2026.1772860

**Published:** 2026-03-09

**Authors:** Awais Paracha, Derek Tran, Margot Noyelle, Jonathan Kapilian, Zohair Siddiqui, Adrian Choppa, Anthony Corsi, Jacob Stone, Xin-Hua Zhu

**Affiliations:** 1 Department of Medicine, Northwell Health, New Hyde Park, NY, United States; 2 Donald and Barbara Zucker School of Medicine at Hofstra/Northwell, Hempstead, NY, United States; 3 Department of Medicine, Thomas Jefferson University, Philadelphia, PA, United States; 4 Department of Medicine, California Pacific Medical Center, San Francisco, CA, United States; 5 Department of Medicine, The University of Tennessee Health Science Center, Memphis, TN, United States; 6 Northwell Health Cancer Institute, Northwell Health, New Hyde Park, NY, United States

**Keywords:** *HRR*, mCRPC, metastatic castrate-resistant prostate cancer, metastatic hormone-sensitive prostate cancer, mHSPC, NGS - next generation sequencing, precision medicine, metastatic prostate cancer

## Abstract

**Background:**

Prostate cancer (PC) is the most common cancer in men in the United States with a 5-year relative survival rate for people with distant metastases of 36%. We conducted a single institution, retrospective cohort study of patients with metastatic PC to investigate whether certain gene mutations can be used as predictive biomarkers.

**Methods:**

200 patients with metastatic hormone sensitive (mHSPC) and castration resistant (mCRPC) prostate cancer who had a FoundationOne report and were treated from June 2007 to October 2024 were included in the study. Disease progression was evaluated according to RECIST criteria, PCWG3 criteria, and PSA values. Assessed gene mutations included *SPOP, p53, Rb, PTEN*, and *HRR* genes. Overall survival (OS) and progression-free survival (PFS) were calculated for mHSPC and mCRPC.

**Results:**

Among 200 patients, there were 182 patients with mHSPC and 174 patients with mCRPC. Average age at diagnosis of metastatic disease was 71.5, ECOG was 0.76, and median PSA was 75.6 ng/mL. 152 patients had high-volume disease. 102 patients passed away. Patients with a *p53* mutation (n=99) had lower OS in mHSPC (50.7 months vs 86.2 months, p<0.01). Patients with a *PTEN* mutation (n=50) had a lower OS in mHSPC (50.6 months vs 65.8 months, p=0.03) and mCRPC (29.5 months vs 46.0 months, p=0.04). Patients with *HRR* mutations (n=43) had lower OS in mHSPC (41.4 months vs 64.6 months, p<0.01) and mCRPC (18.4 months vs 42.8 months, p=0.04). *p53* and *PTEN* mutations were associated with shorter PFS in mCRPC (13.8 months vs 23.2 months, p=0.03; 12.2 months vs 22.6 months, p<0.01, respectively). Mutations in *SPOP* (n=13 mHSPC, n=9 mCRPC) and *RB1* (n=12 mHSPC, n=10 mCRPC) were not associated with statistically significant differences in OS and PFS.

**Conclusions:**

*PTEN*, and *HRR* mutations were associated with shorter OS in both mHSPC and mCRPC. *p53* mutations are associated with shorter OS only in mHSPC. *p53* and *PTEN* mutations were associated with shorter PFS only in mCRPC.

## Introduction

Prostate cancer (PC) is the second leading cause of cancer-related death in men (1).​ For patients with distant metastatic PC, the 5-year survival rate is approximately 37% (2).​ The current standard of care for metastatic hormone sensitive prostate cancer (mHSPC) includes androgen deprivation therapy (ADT), docetaxel, and androgen receptor pathway inhibitors (ARPIs) for high-volume disease, along with combined ADT and ARPIs for high- and low-volume diseases (3).​ The current standard of care for metastatic castrate resistant prostate cancer (mCRPC) includes docetaxel, cabazitaxel, radium-223 and Lu-PSMA-617 (4).​ The standard of care for the first and second lines of precision medicine in mCRPC is PARP inhibitors if there are homologous recombination repair (*HRR*) alterations, including *BRCA1, BRCA2, ATM, ATR, BRIP1, BARD1, CDK12, CHEK1, CHEK2, FANCA, FANCL, MLH1, MRE11A, NBN, PALB2, RAD51B, RAD51C, RAD51D, and RAD54L* (4).

There is interest in expanding real-world data for patients undergoing treatment that have metastatic PC which transforms from hormone sensitive (HS) to hormone resistant (HR) disease. Treatment options and prognosis changes once patients progress to mCRPC, so stratifying patients based on mutation status is beneficial to identify high risk individuals.

We have conducted a retrospective cohort study on metastatic PC patients seen at a tertiary academic cancer center who received next-generation sequencing (NGS) testing on their biopsy or blood samples. Our primary objective was to assess which gene mutations are associated with a more rapid transformation from mHSPC to mCRPC to identify mutations that are related to poor prognosis in patients undergoing treatment for PC. We analyzed overall survival (OS) and progression-free survival (PFS) of these patients, as well as the time elapsed when transitioning from HS to HR disease. We hypothesize that gene mutations in *SPOP, RB1, p53, PTEN*, and *HRR* genes will be associated with HS and HR disease, along with more rapid transition from mHSPC to mCRPC and lower OS and PFS.

## Materials and methods

We conducted a single institution retrospective cohort study of 200 patients diagnosed with metastatic PC from June 2007 to October 2024. Using FoundationOne NGS analysis, we investigated if certain specific gene mutations, including *SPOP, p53, RB1, PTEN*, and *HRR* genes, were associated with differences in OS and PFS (5). Patients with a FoundationOne report and diagnoses of “Prostate Acinar Adenocarcinoma” and “Prostate Cancer (Not otherwise specified)” were included in our study. Patients who had cancer that was not prostatic adenocarcinoma, such as prostate neuroendocrine tumors, were excluded. Patient race, age and Eastern Cooperative Oncology Group (ECOG) performance score at diagnosis, date of death, presence of metastatic disease at initial diagnosis, and locations of metastatic disease were recorded. Treatment data, including the dates of treatment, type of treatment, and treatments prior to the diagnosis of metastatic disease (e.g. prior prostatectomy or radiation therapy) were also recorded. We recorded the patients’ Gleason scores and associated gene mutations, including HRR gene aberrations, *SPOP, p53, RB1*, and *PTEN*. Each patient’s disease was classified as HR or HS based on the treatment course.

We calculated the time from initial diagnosis of metastatic disease to the first progression of disease. We also calculated the time from first progression of disease to the second progression, representing months of progression-free survival. Progression of disease was defined based on PSA trends, osseous progression, and radiographic findings according to the Prostate Cancer Clinical Trials Working Group 3 (PCWG-3) and RECIST 1.1 guidelines (6, 7). Per PCWG-3 guidelines, osseous progression was determined by the 2 + 2 rule. PSA progression was defined by an increase of at least 25% and at least 2 ng/mL increase from nadir, confirmed by a second PSA measurement at least 3 weeks later. Per RECIST 1.1 guidelines, target lesions included measurable soft tissue lesions (at least 10 mm in the longest dimension on CT scan) and lymph nodes (at least 15 mm in short axis on CT scan). Progression of disease was defined as a 20% increase in the sum of the diameter of all target lesions, and at least a minimum increase of 5 mm in absolute sum diameter. Measurable lesions were followed in subsequent scans to assess for stable disease, partial or complete response to treatment, or progressive disease.

OS was calculated using the months between initial diagnosis of metastatic PC and date of death. HS PFS was calculated from the time between initial diagnosis and first progression HR disease. HR PFS was calculated from the time between progression to HR disease and the second progression of disease. The Kaplan-Meier method was used to analyze PFS and OS to represent the survival patterns in HR and HS disease, as well as subgroup analysis based on aggressive disease characteristics including high-volume, *de novo* metastatic, and high-grade disease.

## Results

### Cohort characteristics

Two hundred patients were included in our study. Samples were obtained with liquid biopsy (n=137, 68.5%) and tissue biopsy (n=63, 31.5%). 157 (78.5%) of the samples were taken during a mCRPC state, while 43 (21.5%) were taken during a mHSPC state. 90 patients were Caucasian (45%), 55 patients were African American (27.5%), and 55 patients were from another race, including Hispanic or Asian (27.5%). The average age at diagnosis of metastatic disease was 71.5 years. The median and mean Gleason score at diagnosis were 8.5 and 8.3, respectively, based on a sample of 136 patients. Each patient’s median PSA values were recorded, and the average of these median PSAs was 75.6 ng/mL. Average ECOG Performance Status at diagnosis was 0.76.

Among the 200 patients, there were 182 patients (91%) with HS disease and 174 patients (87%) with HR disease. 156 patients (78%) with mHSPC progressed to mCRPC. The average time to first progression of disease was 20.1 months, which represents the number of months the patients were hormone sensitive. For hormone-refractory patients, the average time from the first progression to a second progression was 17.8 months. The median OS for hormone-sensitive (HS) patients was 61.5 months. The median OS for hormone-resistant (HR) patients was 40.7 months. The median progression-free survival (PFS) was 16.7 months for HS patients and 18.8 months for HR patients. 102 patients (51%) passed away or entered hospice care during the study period.

152 patients had high-volume disease (76%), defined by the presence of visceral metastases and/or the presence of at least 4 bone lesions, with at least one beyond the spine or pelvis. 93 patients (46.5%) had *de novo* metastatic disease at initial diagnosis. 101 patients (50.5%) had an International Society of Urological Pathology (ISUP) grade group of 4 or higher. The most common genetic aberrations in our cohort were *p53* (49.5%, 99/200), *PTEN* (25% 50/200), and *HRR* mutations (21.5% 43/200). 56 patients (28%) had more than one mutation; 30 patients (15%) had a *p53/PTEN* mutation and 18 patients (9%) had a *p53/HRR* gene mutation. Prevalence of these mutations is shown in **Table 1**.

**Table 1:** Target genes and frequency in metastatic prostatic adenocarcinoma.

Target gene	Frequency (%)
*p53*	99 (49.5%)
*PTEN*	50 (25%)
*SPOP*	14 (7%)
*RB1*	12 (6%)
*HRR* genes	43 (21.5%)
*BRCA1*	*3 (1.5%)*
*BRCA2*	*13 (6.5%)*
*ATM*	*2 (1%)*
*ATR*	*2 (1%)*
*BARD1*	*1 (0.5%)*
*CDK12*	*7 (3.5%)*
*CHEK2*	*1 (0.5%)*
*FANCA*	*3 (1.5%)*
*MLH1*	*4 (2%)*
*MRE11A*	*1 (0.5%)*
*NBN*	*2 (1%)*
*PALB2*	*1 (0.5%)*
*RAD51C*	*1 (0.5%)*
*RAD54L*	*2 (1%)*
*BRIP1, CHEK1, FANCL, RAD51B, RAD51D*	*0 (0%)*
*p53* and *PTEN*	30 (15%)
*p53* and *HRR* genes	18 (9%)
*p53* and *RB1*	9 (4.5%)
*p53* and *SPOP*	7 (3.5%)

Aggressive clinical phenotypes (high-volume disease, *de novo* metastatic disease, and ISUP grade group 4 or 5) were compared between patients with and without the aforementioned genomic alterations. Chi-square analysis revealed no statistically significant differences in the distribution of high- versus low- volume disease, *de novo* presentation, or high Gleason grade in *p53*, *PTEN* and *HRR* gene mutations (all p>0.15).

189 patients (94.5%) received treatment with ARPIs including abiraterone, enzalutamide, apalutamide, and darolutamide. 99 patients (49.5%) received treatment with taxane chemotherapy, including cabazitaxel and docetaxel. 21 patients (10.5%) received treatment with PARP inhibitors, including olaparib, talazoparib, and rucaparib. Distribution of additional treatments, such as GnRH agonists, additional-line chemotherapy, and radiation are shown in **Table 2**.

**Table 2:** Targeted treatments and frequency in metastatic prostatic adenocarcinoma.

Treatment	Frequency (%)
Second generation anti-androgen agents (Abiraterone, Enzalutamide, Aplatumide, Darolutamide)	189 (94.5%)
Taxane chemotherapy (Cabazitaxel, Docetaxel, Paclitaxel)	99 (49.5%)
Second-line chemotherapy	40 (20%)
Third-line chemotherapy	11 (5.5%)
PARP inhibitor (Olaparib, Nucaparib, Teloparib, Rucaparib)	21 (10.5%)
Lutetium Lu-177 vipivotide tetraxetan	5 (2.5%)
Radium-223	17 (8.5%)
Sipuleucel-T	26 (13%)
Radiation (Brachytherapy, stereotactic radiosurgery)	84 (42%)
Radical prostatectomy	46 (23%)

### Tumor suppressor gene associations with OS and PFS

**Figure 1:**
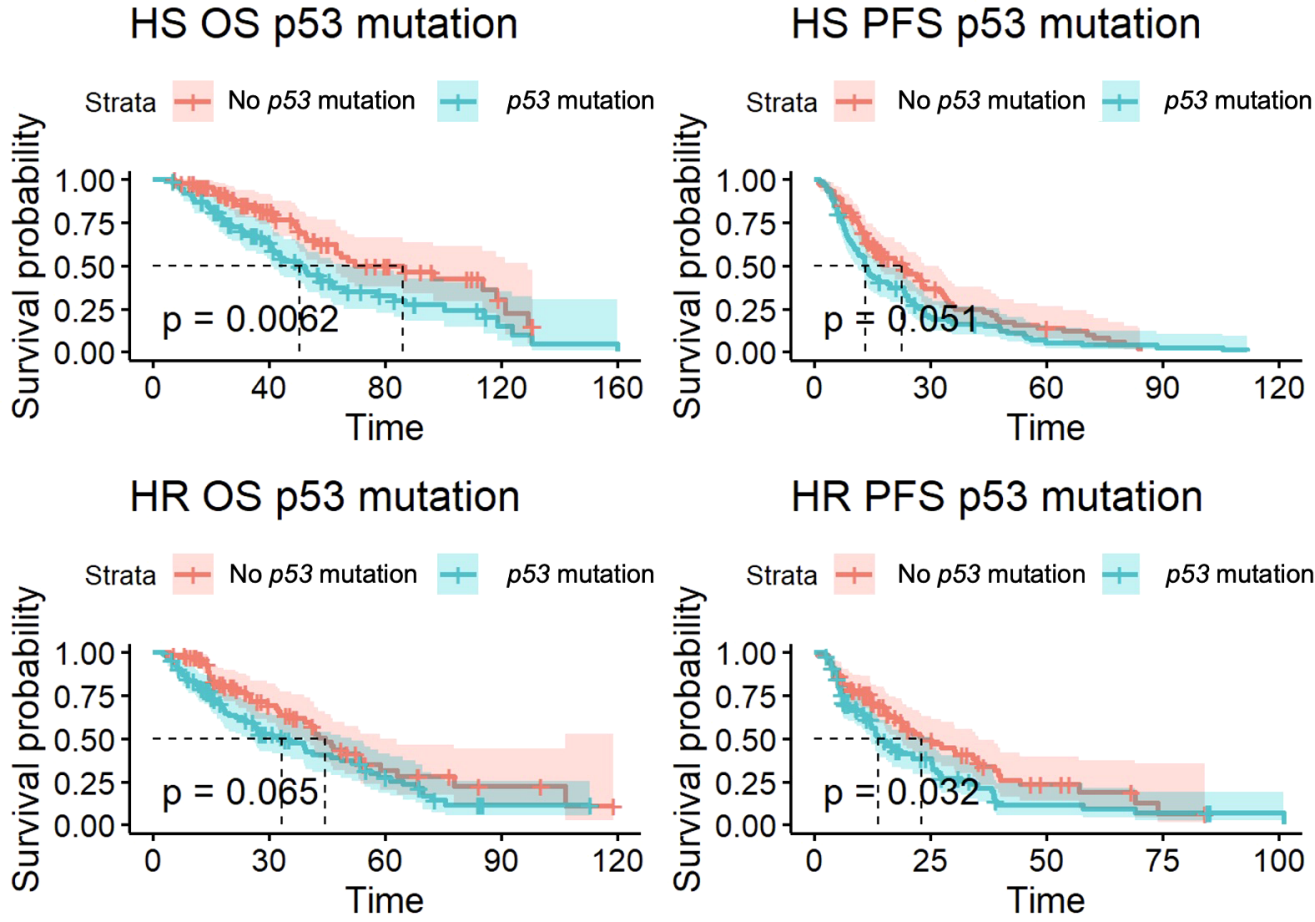
Overall survival (OS) and progression-free survival (PFS) for *p53* mutant patients with hormone-sensitive (HS) and hormone-resistant (HR) metastatic prostate cancer.

*p53* and *PTEN* mutations were associated with statistically significant differences in overall survival (OS) and progression-free survival (PFS). 99 patients had a *p53* mutation, with 92 HS and 93 HR patients. Of patients with a *p53* mutation, 86 had mHSPC that developed into mCRPC. As shown in **Figure 1**, there was a significantly lower OS for patients with mHSPC (50.7 months vs 86.2 months, p<0.01). For mCRPC patients, median OS in patients with *p53* mutations was 33.3 months compared to 44.5 months in non-*p53* mutants (p=0.07). There was also a shorter PFS in patients with mCRPC (13.8 months vs 23.2 months, p=0.03).

**Figure 2:**
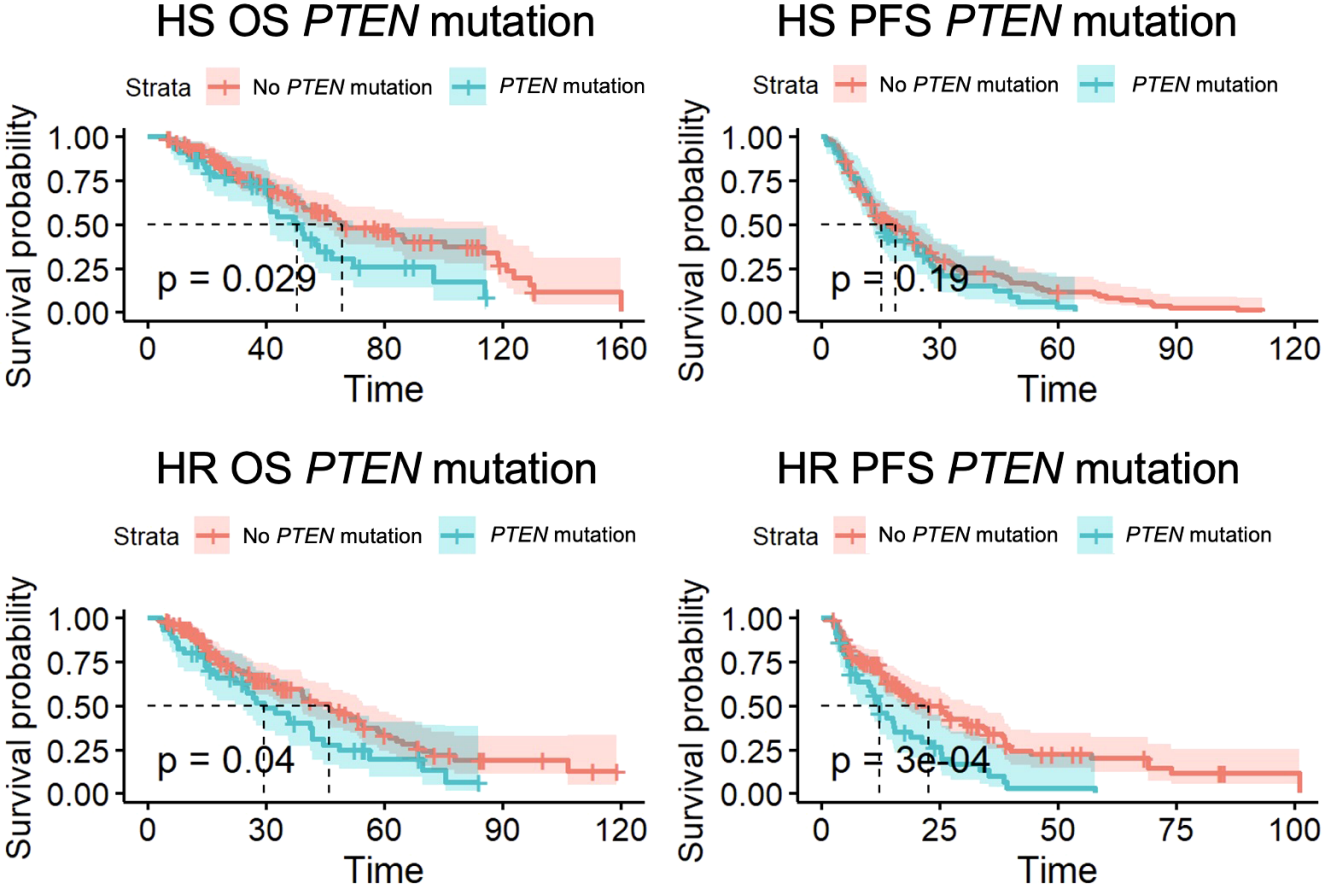
Overall survival (OS) and progression-free survival (PFS) for *PTEN* mutant patients with hormone-sensitive (HS) and hormone-resistant (HR) metastatic prostate cancer.

50 patients had a *PTEN* mutation, with 46 HS patients and 45 HR patients. 41 patients with a *PTEN* mutation had mHSPC that developed into mCRPC. As shown in **Figure 2**, for patients with a *PTEN* mutation, there was a significantly lower OS for patients with both HS (50.6 months vs 65.8 months, p=0.03) and HR cancer (29.5 months vs 46.0 months, p=0.04). PFS was also shorter for patients with mCRPC (12.2 months vs 22.6 months, p<0.01).

**Figure 3:**
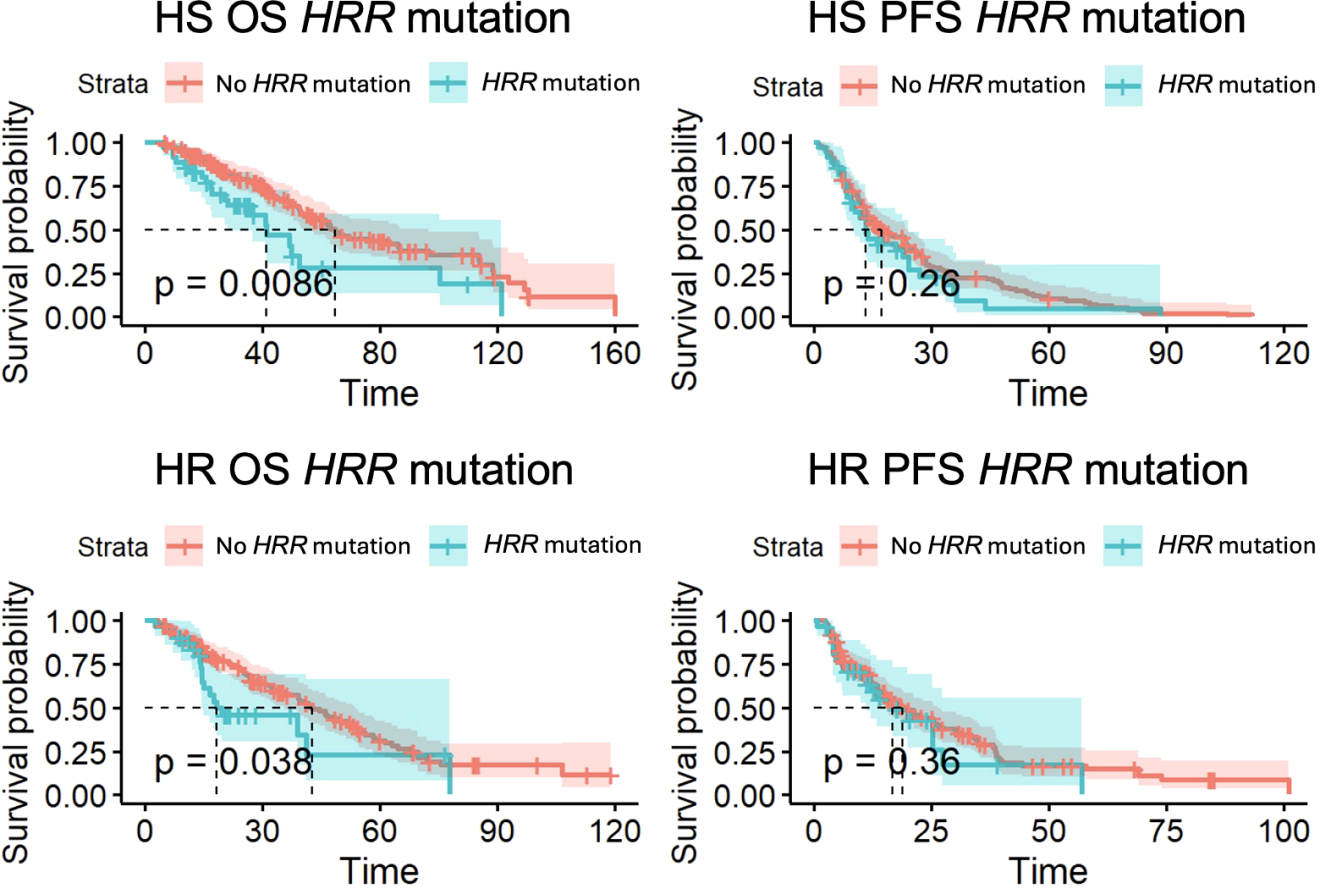
Overall survival (OS) and progression-free survival (PFS) for *HRR* mutant patients with hormone-sensitive (HS) and hormone-resistant (HR) metastatic prostate cancer.

*HRR* mutations were associated with statistically significant differences in OS. 43 patients had *HRR* mutations, with 36 mHSPC and 32 mCRPC patients. 31 *HRR* mutant patients had mHSPC that developed into mCRPC. OS was significantly lower for both HS patients (41.4 months vs 64.6 months, p<0.01) and HR patients (18.4 months vs 42.8 months, p=0.04) (**Figure 3**). There was no significant difference in PFS for HS patients (p=0.26) and HR patients (p=0.36) with *HRR* aberrations.

Given the presence of 56 co-mutations in our cohort, we further compared survival outcomes in *p53/HRR-*mutated and *p53/PTEN*-mutated patients (**Supplementary Figures 1-S4**). Patients with a *p53* mutation and either *HRR* or *PTEN* mutations consistently displayed the poorest OS in both HR and HS disease (all p<0.01) compared to patients with single mutations or wild-type genes (**Supplementary Table 1**).

For patients with *SPOP* mutations, there was no statistically significant difference in OS among HS and HR patients (n=13 mHSPC, n=9 mCRPC, p=0.51, p=0.46, respectively). There was also no significant difference in PFS in HS and HR patients (p=0.28, p=0.50, respectively). Similarly, patients with *RB1* mutations had no significant difference in OS for HS and HR patients (n=12 mHSPC, n=10 mCRPC, p=0.08, 0.10, respectively) or PFS (p=0.41, p=0.07, respectively).

## Discussion

In this retrospective cohort study of two hundred patients with metastatic prostate cancer, we found that mutations in *PTEN* and *HRR* genes were associated with significantly worse overall survival (OS) in mHSPC and mCRPC. p53 mutations was associated with worse OS only in mHSPC. Mutations in *p53* and *PTEN* were also associated with shorter progression-free survival (PFS) in patients with mCRPC. Results for *SPOP* and *RB1* mutations were inconclusive due to a low sample size. These findings underscore the predictive value of specific tumor gene alterations in metastatic prostate cancer and contribute to the growing body of evidence on genetic drivers of disease progression.

Prior studies have demonstrated that alterations in tumor suppressor genes, including *p53, PTEN*, and *RB1* are associated with androgen insensitivity and aggressive disease in mHSPC (8). Our study provides further insights onto the prognostic role of these mutations in mCRPC, as well as the transition between mHSPC and mCRPC. Furthermore, insights from NGS may improve prognostic ability. In this cohort, *p53*, *PTEN*, and *HRR* mutations were not significantly associated with known markers of aggressive disease, including high-volume disease, *de novo* presentation, and higher ISUP grade. Although formal multivariable modeling was not performed due to sample size limitations, our data suggest that genetic alterations do not merely track with aggressive characteristics, and may represent distinct drivers of disease progression.

*p53* is a key regulator of genomic stability and apoptosis; loss of function facilitates tumor progression and therapeutic resistance (9, 10). In our cohort, patients with *p53* mutations experienced markedly shorter OS in the hormone-sensitive state. We also found shorter PFS in patients with hormone-resistant cancer and *p53* mutations. These findings are consistent with earlier reports that highlight *p53* as a critical determinant of treatment resistance and rapid disease progression (9, 11, 12). Although not statistically significant, *p53* mutations were also associated with shorter OS in castration-resistant states (p=0.07). Moreover, co-mutations including *p53* and either *HRR* or *PTEN* had worse outcomes in both mHSPC and mCRPC compared to any single mutation, emphasizing the role of *p53* dysfunction in aggressive disease.

*PTEN* loss has also been extensively implicated in prostate cancer pathogenesis through activation of the PI3K/AKT pathway which promotes tumor growth and survival (13–15). In our study, patients with *PTEN* mutations had significantly shorter OS, and patients with hormone resistant cancer also had shorter PFS. This supports previous literature that identifies *PTEN* loss as both a predictive biomarker of poor response to androgen receptor (AR)-targeted therapy and an indicator of aggressive disease biology (14, 16–19). These data further suggest that dual inhibition strategies targeting both AR signaling and PI3K/AKT pathways may be especially relevant in this subset. A Phase III trial determined that patients with *PTEN-*deficient mHSPC had a statistically significant improvement in PFS while on a dual regimen of capivasertib, a PI3K/AKT pathway inhibitor, and abiraterone (20). Another Phase I trial demonstrated that a PI3K/AKT inhibitor in combination with docetaxel was well tolerated in treating *PTEN*-mutated solid tumors, albeit with limited efficacy (21).

The *HRR* mutation group, which included alterations in *BRCA1/2, PALB2, ATM, CDK12*, and other DNA repair genes, also demonstrated significantly worse survival outcomes compared with wild-type counterparts. These genes are involved in the repair of breaks in the DNA strands which are required for cell replication. In the absence of properly functioning homologous recombination repair genes, cells resort to less accurate methods of repairing DNA double-stranded breaks. Additional mutations may accumulate and drive uncontrolled cell division, leading to more aggressive cancer proliferation (22). While PARP inhibitors have emerged as effective, targeted therapies for *BRCA1/2* and other *HRR*-mutated cancers, only a minority of patients in our cohort received PARP inhibitors, which may partly explain the poor outcomes observed (23–29). These findings highlight the clinical importance of timely genomic testing and treatment stratification, especially as PARP inhibitors and combination strategies gain broader regulatory approval.

Mutations in *SPOP* and *RB1* did not show a statistically significant effect on survival in our cohort, despite prior studies suggesting their potential roles in prostate cancer biology (9, 11, 30). The relatively small number of patients with *SPOP* and *RB1* mutations in our cohort limited the statistical power to detect meaningful differences.

Our findings reinforce the clinical utility of genomic profiling in metastatic prostate cancer, not only for guiding targeted therapy but also for risk stratification. Second, the consistent association of *p53, PTEN*, and *HRR* mutations with poor outcomes emphasizes the need for prospective trials testing tailored treatment approaches, such as early incorporation of PARP inhibitors or PI3K pathway inhibitors, in patients with these gene alterations (31). Third, we found that mutations in *p53* and *PTEN* were associated with shorter progression-free survival (PFS) in patients with hormone-resistant cancer. Recent studies have linked *p53* and *PTEN* mutations with the development of hormone-resistant cancer, and our finding suggests that these mutations may affect cancerous cells through mechanisms beyond just the development of hormone resistance (32, 33).

Our study is limited by its retrospective design, which is inherently subject to selection bias and incomplete data capture. Gene testing was performed at varying time points in the disease course and tumor heterogeneity which may have influenced observed associations. For instance, a *p53/PTEN/HRR* mutation in mHSPC would confer different prognostic meaning than a comparable mutation detected in mCRPC. Genomic studies demonstrate that tumor suppressor genes like *TP53* and *PTEN* are present in 28-37% of mHSPC and are further enriched in mCRPC (67-73%), suggesting approximately 50% concordance from mHSPC to mCRPC (34, 35). In our study population, biopsies were only retrieved early in the diagnosis of mCRPC as routine standard of care. This would further increase concordance beyond reported values, as compared to NGS of late-stage mCRPC which would presumably have had more time to accumulate deleterious mutations. Furthermore, our data still shows significant differences in OS between wild-type and mutated *p53, PTEN*, and *HRR* genes within mHSPC. This suggests that NGS analysis in an early mCRPC setting may reflect molecular changes in mHSPC.

Our study also compares the length of progression-free and overall survival for patients with several different gene mutations and subgroups. The number of variables considered and limited sample size increases the risk of a type I error. Furthermore, while our sample size was larger than some prior institutional series, the number of patients within specific mutation subgroups remained modest, restricting the generalizability of subgroup analyses. For instance, we found no statistically significant difference in OS for mCRPC patients with *p53* mutations (p=0.07), despite its established role in prostate cancer progression (36). *p53* inactivation has been associated with poorer responses to ARPIs like abiraterone and enzalutamide (33). However, other studies have demonstrated no significant association of *p53* mutations with OS, so further research is required to clarify the role of *p53* mutations in mCRPC prognostication (37).

Despite these limitations, our study contributes to the evidence that alterations in tumor suppressor genes, particularly in *p53, PTEN*, and *HRR* genes, are key predictive markers in metastatic prostate cancer as HS disease becomes HR disease (9, 11, 16–18, 38, 39). Future research should focus on integrating genomic data with clinical risk models, validating these associations in larger multi-institutional cohorts, and evaluating precision-medicine strategies that address the biological vulnerabilities conferred by these mutations.

## Conclusion

In this single-institution retrospective cohort study, we found that mutations in *PTEN* and *HRR* genes were significantly associated with shorter overall survival in mHSPC and mCRPC. *p53* mutation was associated with lower overall survival in mHSPC only. Additionally, *p53* and *PTEN* mutations conferred shorter progression-free survival in patients with mCRPC. These findings highlight the importance of genomic profiling for risk stratification and underscore the need for prospective studies testing targeted therapeutic approaches and potential predictive biomarkers.

## Data Availability

The raw data supporting the conclusions of this article will be made available by the authors, without undue reservation.

## References

[1] Henley SJ Ward EM Scott S Ma J Anderson RN Firth AU . Annual report to the nation on the status of cancer, part I: National cancer statistics. Cancer. (2020) 126:2225–49. doi: 10.1002/cncr.32802, PMID: 32162336 PMC7299151

[2] Raychaudhuri R Lin DW Montgomery RB . Prostate cancer: A review. JAMA. (2025) 333:1433–46. doi: 10.1001/jama.2025.0228, PMID: 40063046

[3] Schaeffer EM Srinivas S Adra N An Y Barocas D Bitting R . Prostate cancer, version 4.2023, NCCN clinical practice guidelines in oncology. J Natl Compr Canc Netw. (2023) 21:1067–96. doi: 10.6004/jnccn.2023.0050, PMID: 37856213

[4] Hussain M Fizazi K Shore ND Heidegger I Smith MR Tombal B . Metastatic hormone-sensitive prostate cancer and combination treatment outcomes: A review. JAMA Oncol. (2024) 10:807–20. doi: 10.1001/jamaoncol.2024.0591, PMID: 38722620

[5] Woodhouse R Li M Hughes J Delfosse D Skoletsky J Ma P . Clinical and analytical validation of FoundationOne Liquid CDx, a novel 324-Gene cfDNA-based comprehensive genomic profiling assay for cancers of solid tumor origin. PloS One. (2020) 15:e0237802. doi: 10.1371/journal.pone.0237802, PMID: 32976510 PMC7518588

[6] Scher HI Morris MJ Stadler WM Higano C Basch E Fizazi K . Trial design and objectives for castration-resistant prostate cancer: updated recommendations from the prostate cancer clinical trials working group 3. J Clin Oncol. (2016) 34:1402–18. doi: 10.1200/JCO.2015.64.2702, PMID: 26903579 PMC4872347

[7] Eisenhauer EA Therasse P Bogaerts J Schwartz LH Sargent D Ford R . New response evaluation criteria in solid tumours: revised RECIST guideline (version 1.1). Eur J Cancer. (2009) 45:228–47. doi: 10.1016/j.ejca.2008.10.026, PMID: 19097774

[8] Pedrani M Salfi G Merler S Testi I Agrippina Clerici CM Pecoraro G . Integrating aggressive-variant prostate cancer-associated tumor suppressor gene status with clinical variables to refine prognosis and predict androgen receptor pathway inhibitor response in metastatic hormone-sensitive setting. Int J Mol Sci. (2025) 26. doi: 10.3390/ijms26115309, PMID: 40508118 PMC12154572

[9] Zhou J Lai Y Peng S Tang C Chen Y Li L . Comprehensive analysis of TP53 and SPOP mutations and their impact on survival in metastatic prostate cancer. Front Oncol. (2022) 12:957404. doi: 10.3389/fonc.2022.957404, PMID: 36119488 PMC9471084

[10] Xue L Han X Liu R Wang Z Li H Chen Q . MDM2 and P53 polymorphisms contribute together to the risk and survival of prostate cancer. Oncotarget. (2016) 7:31825–31. doi: 10.18632/oncotarget.3923, PMID: 26025918 PMC5077979

[11] Mateo J Seed G Bertan C Rescigno P Dolling D Figueiredo I . Genomics of lethal prostate cancer at diagnosis and castration resistance. J Clin Invest. (2020) 130:1743–51. doi: 10.1172/JCI132031, PMID: 31874108 PMC7108902

[12] De Laere B Oeyen S Mayrhofer M Whitington T van Dam PJ Van Oyen P . TP53 outperforms other androgen receptor biomarkers to predict abiraterone or enzalutamide outcome in metastatic castration-resistant prostate cancer. Clin Cancer Res. (2019) 25:1766–73. doi: 10.1158/1078-0432.CCR-18-1943, PMID: 30209161 PMC6330086

[13] Kulasegaran T Oliveira N . Metastatic castration-resistant prostate cancer: advances in treatment and symptom management. Curr Treat Options Oncol. (2024) 25:914–31. doi: 10.1007/s11864-024-01215-2, PMID: 38913213 PMC11236885

[14] Gupta S To TM Graf R Kadel EE 3rd Reilly N Albarmawi H . Real-world overall survival and treatment patterns by PTEN status in metastatic castration-resistant prostate cancer. JCO Precis Oncol. (2024) 8:e2300562. doi: 10.1200/PO.23.00562, PMID: 38547419 PMC10994466

[15] Choudhury AD . PTEN-PI3K pathway alterations in advanced prostate cancer and clinical implications. Prostate. (2022) 82 Suppl 1:S60–72. doi: 10.1002/pros.24372, PMID: 35657152

[16] Antar A Xia Y Al-Mistarehi AH Papali P Alfonzo Horowitz M Sriram S . PTEN mutations associated with increased recurrence and decreased survival in patients with prostate cancer spinal metastasis. Curr Oncol. (2025) 32:1–14. doi: 10.3390/curroncol32060331, PMID: 40558274 PMC12192451

[17] Repka MC Sholklapper T Zwart AL Danner M Ayoob M Yung T . Prognostic utility of biopsy-based PTEN and ERG status on biochemical progression and overall survival after SBRT for localized prostate cancer. Front Oncol. (2024) 14:1381134. doi: 10.3389/fonc.2024.1381134, PMID: 38585005 PMC10995255

[18] Ferraldeschi R Nava Rodrigues D Riisnaes R Miranda S Figueiredo I Rescigno P . PTEN protein loss and clinical outcome from castration-resistant prostate cancer treated with abiraterone acetate. Eur Urol. (2015) 67:795–802. doi: 10.1016/j.eururo.2014.10.027, PMID: 25454616 PMC4410287

[19] Lotan TL Wei W Morais CL Hawley ST Fazli L Hurtado-Coll A . PTEN loss as determined by clinical-grade immunohistochemistry assay is associated with worse recurrence-free survival in prostate cancer. Eur Urol Focus. (2016) 2:180–8. doi: 10.1016/j.euf.2015.07.005, PMID: 27617307 PMC5014432

[20] Fizazi K Clarke NW De Santis M Uemura H Fay AP Karadurmus N . Capivasertib plus abiraterone in PTEN-deficient metastatic hormone-sensitive prostate cancer: CAPItello-281 phase III study. Ann Oncol. (2025) 37:53–68. doi: 10.1016/j.annonc.2025.10.004, PMID: 41120017

[21] Schram AM Takebe N Chen A Zhou Q Iasonos A Silber J . A phase I study of AZD8186 in combination with docetaxel in patients with PTEN-mutated or PIK3CB-mutated advanced solid tumors. ESMO Open. (2025) 10:105569. doi: 10.1016/j.esmoop.2025.105569, PMID: 40939237 PMC12529303

[22] Incorvaia L Bazan Russo TD Gristina V Perez A Brando C Mujacic C . The intersection of homologous recombination (HR) and mismatch repair (MMR) pathways in DNA repair-defective tumors. NPJ Precis Oncol. (2024) 8:190. doi: 10.1038/s41698-024-00672-0, PMID: 39237751 PMC11377838

[23] Wenzel M Hoeh B Koll F Humke C Fassl A Reis H . Impact of homologous recombination repair/BReast CAncer (BRCA) gene alterations on survival in a real-world setting of metastatic prostate cancer. BJU Int. (2025) 135:117–24. doi: 10.1111/bju.16462, PMID: 38982928 PMC11628939

[24] Grewal K Dorff TB Mukhida SS Agarwal N Hahn AW . Advances in targeted therapy for metastatic prostate cancer. Curr Treat Options Oncol. (2025) 26:465–75. doi: 10.1007/s11864-025-01323-7, PMID: 40299225

[25] Hussain M Kocherginsky M Agarwal N Adra N Zhang J Paller CJ . Abiraterone, olaparib, or abiraterone + Olaparib in first-line metastatic castration-resistant prostate cancer with DNA repair defects (BRCAAway). Clin Cancer Res. (2024) 30:4318–28. doi: 10.1158/1078-0432.CCR-24-1402, PMID: 39115414

[26] Fallah J Xu J Weinstock C Gao X Heiss BL Maguire WF . Efficacy of poly(ADP-ribose) polymerase inhibitors by individual genes in homologous recombination repair gene-mutated metastatic castration-resistant prostate cancer: A US food and drug administration pooled analysis. J Clin Oncol. (2024) 42:1687–98. doi: 10.1200/JCO.23.02105, PMID: 38484203 PMC11095872

[27] Messina C Giunta EF Signori A Rebuzzi SE Banna GL Maniam A . Combining PARP inhibitors and androgen receptor signalling inhibitors in metastatic prostate cancer: A quantitative synthesis and meta-analysis. Eur Urol Oncol. (2024) 7:179–88. doi: 10.1016/j.euo.2023.07.013, PMID: 37574390

[28] Taylor AK Kosoff D Emamekhoo H Lang JM Kyriakopoulos CE . PARP inhibitors in metastatic prostate cancer. Front Oncol. (2023) 13:1159557. doi: 10.3389/fonc.2023.1159557, PMID: 37168382 PMC10165068

[29] de Bono J Mateo J Fizazi K Saad F Shore N Sandhu S . Olaparib for metastatic castration-resistant prostate cancer. N Engl J Med. (2020) 382:2091–102. doi: 10.1056/NEJMoa1911440, PMID: 32343890

[30] Zhang H Jin X Huang H . Deregulation of SPOP in cancer. Cancer Res. (2023) 83:489–99. doi: 10.1158/0008-5472.CAN-22-2801, PMID: 36512624

[31] Pedrani M Barizzi J Salfi G Nepote A Testi I Merler S . The emerging predictive and prognostic role of aggressive-variant-associated tumor suppressor genes across prostate cancer stages. Int J Mol Sci. (2025) 26:1–38. doi: 10.3390/ijms26010318, PMID: 39796175 PMC11719667

[32] Das GM Oturkar CC Menon V . Interaction between estrogen receptors and p53: A broader role for tamoxifen? Endocrinology. (2025) 166:1–12. doi: 10.1210/endocr/bqaf020, PMID: 39891710 PMC11837209

[33] Maughan BL Guedes LB Boucher K Rajoria G Liu Z Klimek S . p53 status in the primary tumor predicts efficacy of subsequent abiraterone and enzalutamide in castration-resistant prostate cancer. Prostate Cancer Prostatic Dis. (2018) 21:260–8. doi: 10.1038/s41391-017-0027-4, PMID: 29302046

[34] Hamid AA Gray KP Shaw G MacConaill LE Evan C Bernard B . Compound genomic alterations of TP53, PTEN, and RB1 tumor suppressors in localized and metastatic prostate cancer. Eur Urol. (2019) 76:89–97. doi: 10.1016/j.eururo.2018.11.045, PMID: 30553611

[35] Velez MG Kosiorek HE Egan JB McNatty AL Riaz IB Hwang SR . Differential impact of tumor suppressor gene (TP53, PTEN, RB1) alterations and treatment outcomes in metastatic, hormone-sensitive prostate cancer. Prostate Cancer Prostatic Dis. (2022) 25:479–83. doi: 10.1038/s41391-021-00430-4, PMID: 34294873 PMC9385473

[36] Ofner H Kramer G Shariat SF Hassler MR . TP53 deficiency in the natural history of prostate cancer. Cancers (Basel). (2025) 17:1–15. doi: 10.3390/cancers17040645, PMID: 40002239 PMC11853097

[37] Abida W Cyrta J Heller G Prandi D Armenia J Coleman I . Genomic correlates of clinical outcome in advanced prostate cancer. Proc Natl Acad Sci U S A. (2019) 116:11428–36. doi: 10.1073/pnas.1902651116, PMID: 31061129 PMC6561293

[38] Vietri MT D’Elia G Caliendo G Resse M Casamassimi A Passariello L . Hereditary prostate cancer: genes related, target therapy and prevention. Int J Mol Sci. (2021) 22:1–17. doi: 10.3390/ijms22073753, PMID: 33916521 PMC8038462

[39] Olmos D Lorente D Alameda D Cattrini C Romero-Laorden N Lozano R . Treatment patterns and outcomes in metastatic castration-resistant prostate cancer patients with and without somatic or germline alterations in homologous recombination repair genes. Ann Oncol. (2024) 35:458–72. doi: 10.1016/j.annonc.2024.01.011, PMID: 38417742

