# Supplementary material for: Real-world precision medicine data in metastatic prostate cancer — a retrospective cohort study
Source: Front Oncol. 2026 Mar 9;16:1772860. doi: 10.3389/fonc.2026.1772860 (PMC13006266; doi:10.3389/fonc.2026.1772860)

|  | **mHSPC OS** | **mHSPC PFS** | **mCRPC OS** | **mCRPC PFS** |
| --- | --- | --- | --- | --- |
| ***p53* WT** | 86.2 | 22.7 | 67.0 | 23.2 |
| ***p53* mutation only** | 65.8 | 14.6 | 65.8 | 16.8 |
| ***HRR* WT** | 64.6 | 17.2 | 63.0 | 18.8 |
| ***HRR* mutation only** | 36.8 | 20.8 | 43.3 | 20.0 |
| ***PTEN* WT** | 65.8 | 18.8 | 67.0 | 22.6 |
| ***PTEN* mutation only** | 69.5 | 17.2 | 56.8 | 15.2 |
| ***HRR/p53* mutation** | 27.1 | 8.5 | 34.0 | 25.3 |
| ***PTEN/p53* mutation** | 40.8 | 12.7 | 43.7 | 11.1 |

**Supplemental Table S1.** Months of overall survival (OS) and progression-free survival (PFS) for *p53, HRR,* and *PTEN*-mutated patients with mHSPC and mCRPC.

**Supplemental Figure S1.** Overall survival (OS) and progression-free survival (PFS) for *p53* and *HRR* co-mutant patients with mHSPC and mCRPC compared to *p53* mutation only and *p53* wild-type (WT).


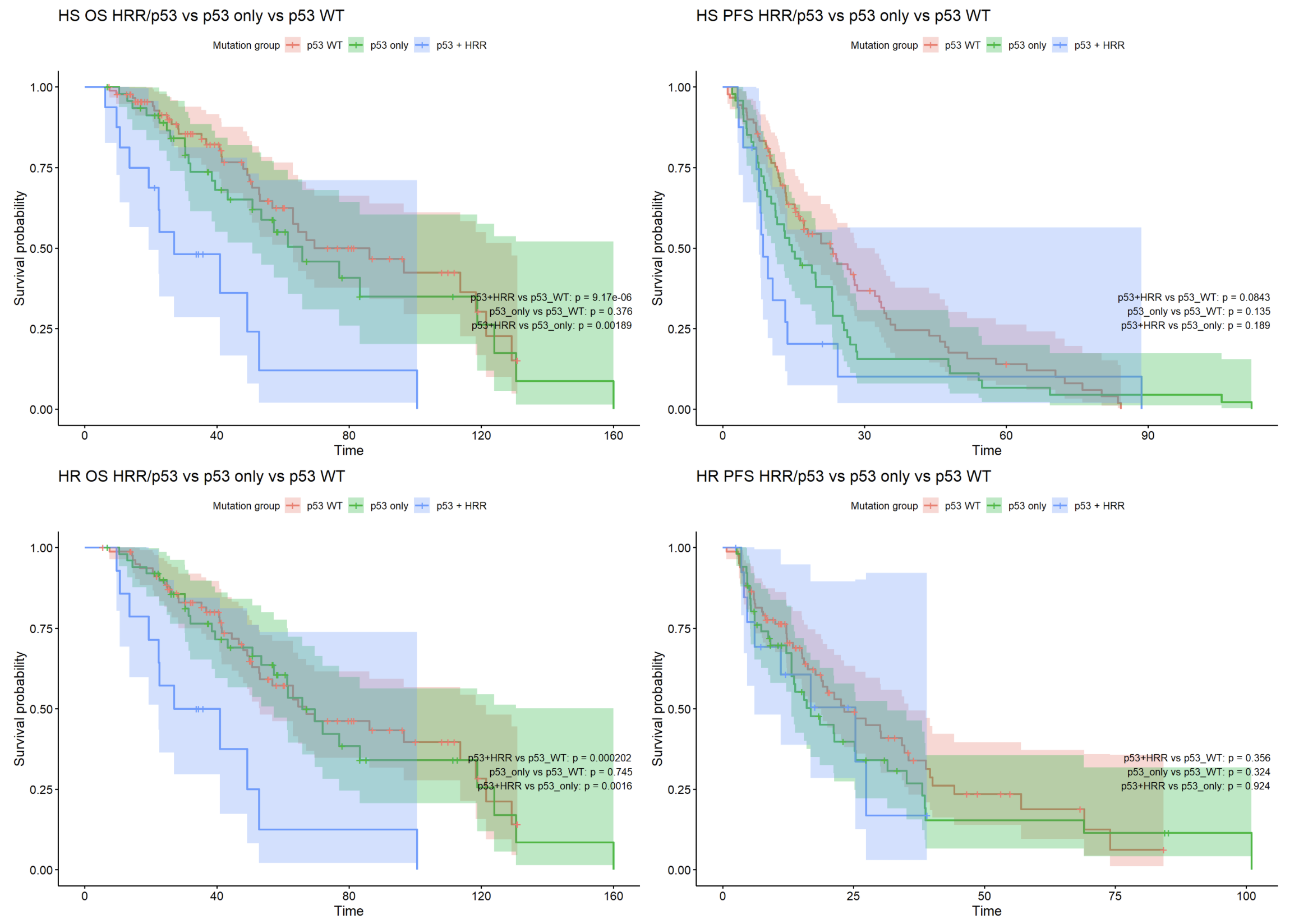


**Supplemental Figure S2.** Overall survival (OS) and progression-free survival (PFS) for *p53* and *HRR* co-mutant patients with mHSPC and mCRPC compared to *HRR* mutation only and *HRR* wild-type (WT).


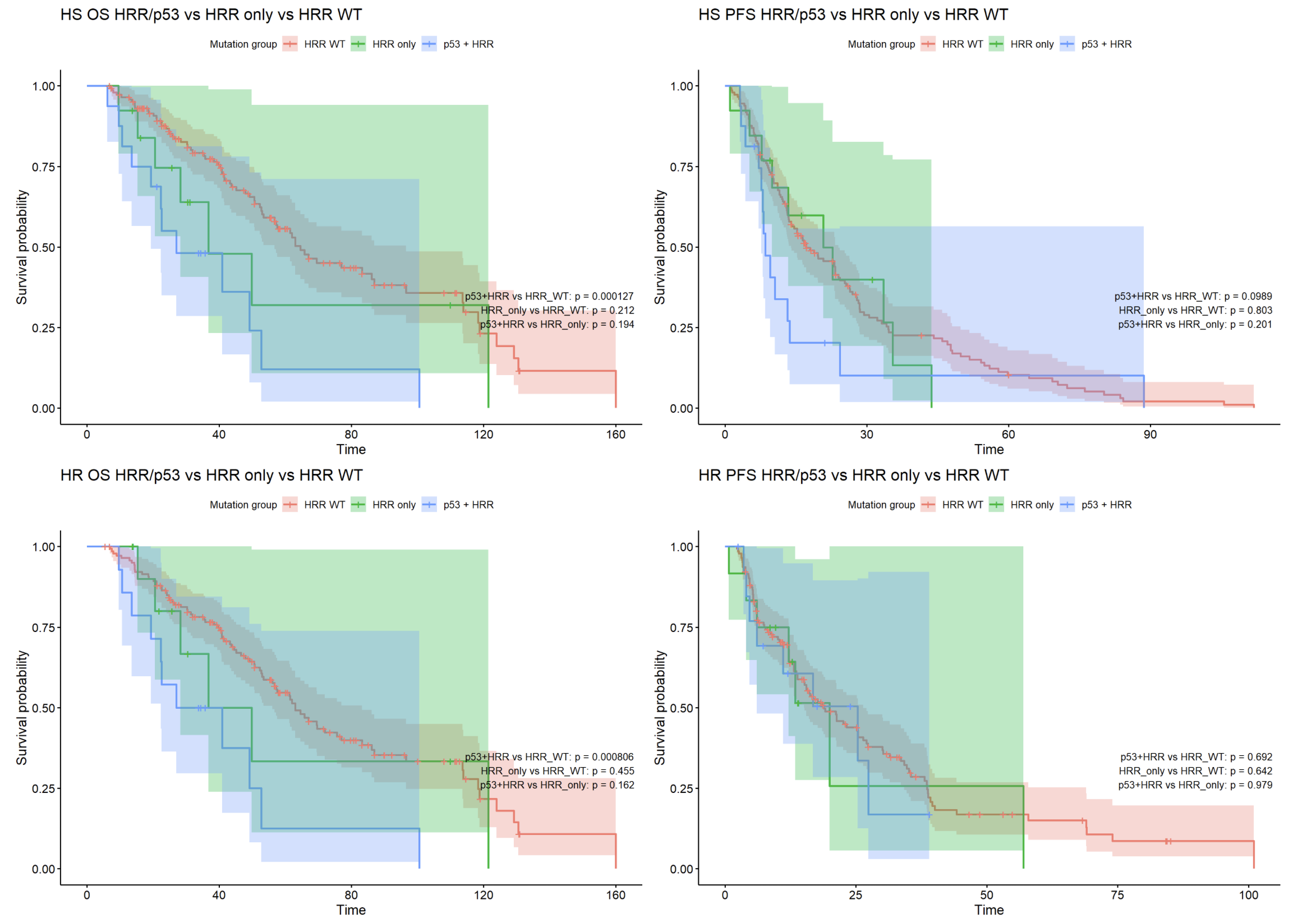


**Supplemental Figure S3.** Overall survival (OS) and progression-free survival (PFS) for *p53* and *PTEN* co-mutant patients with with mHSPC and mCRPC compared to *p53* mutation only and *p53* wild-type (WT).


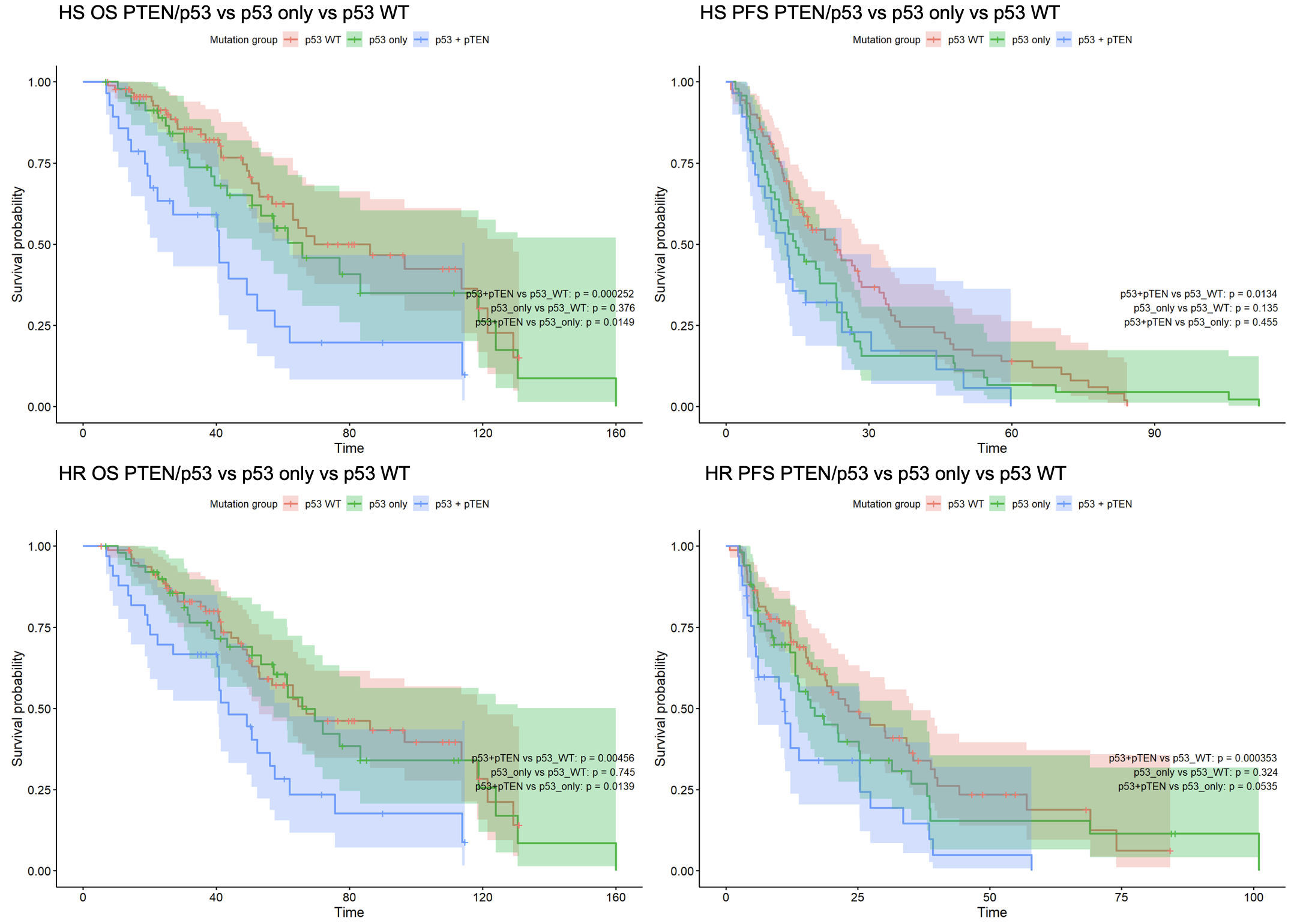


**Supplemental Figure S4.** Overall survival (OS) and progression-free survival (PFS) for *p53* and *PTEN* co-mutant patients with mHSPC and mCRPC compared to *PTEN* mutation only and *PTEN* wild-type (WT).


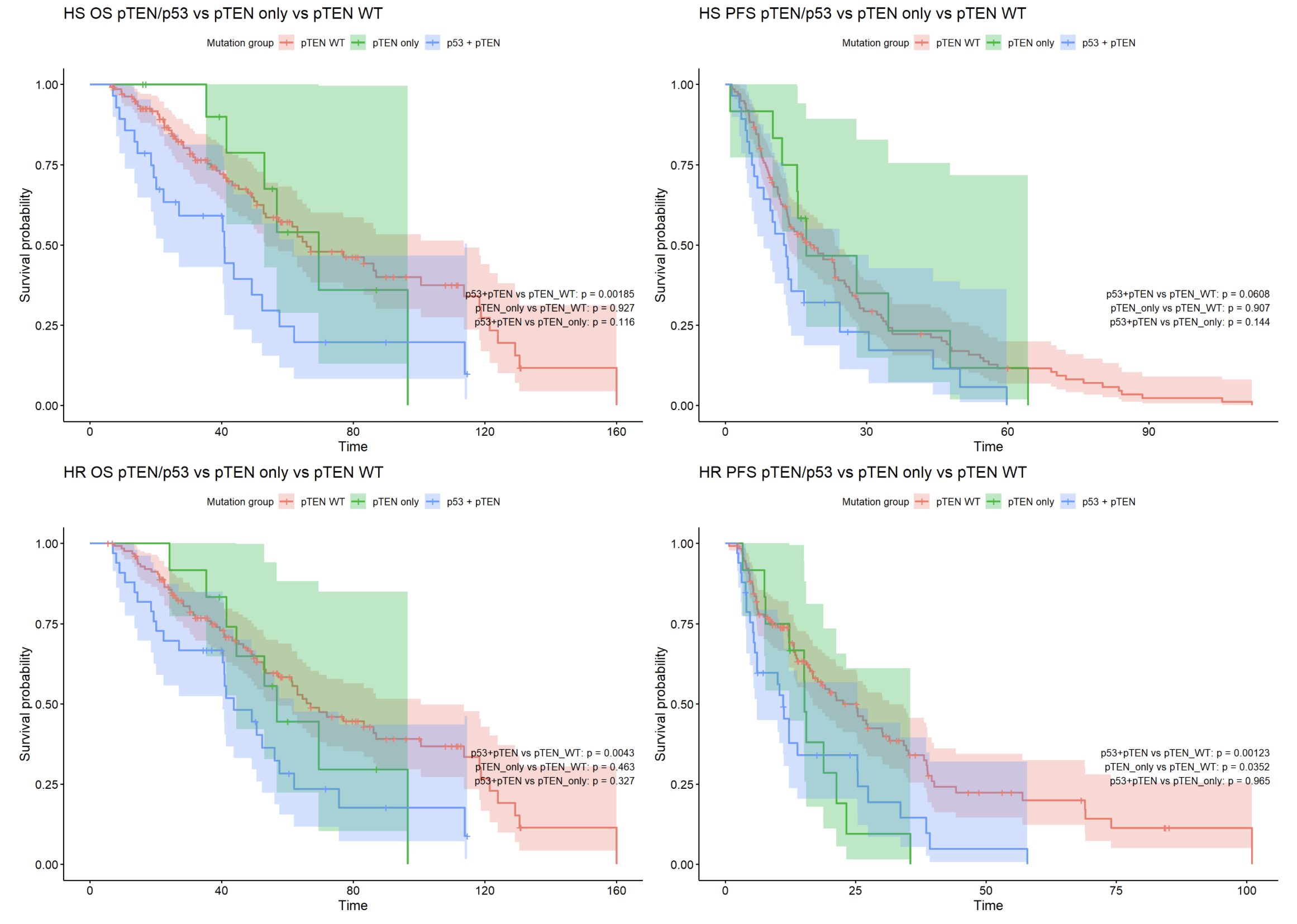

Supplement: Supplementary file 1 [file DataSheet1.docx]
